# Review Department

**Published:** 1889-10

**Authors:** 


### REVIEW DEPARTMENT.

---

I. U. S. Department of Agriculture. Hog Cholera, its History, Nature and Treatment, as determined by the inquiries and investigations of the Bureau of Animal Industry, 1889.

II. Original Investigations of Cattle Diseases in Nebraska, 1886–1888. By Frank S. Billings. Bulletins 7, 8, 9 and 10. Lincoln, Nebraska.

III. D. E. Salmon, Swine-Plague, Hog Cholera, 1887. Reviewed by Frank S. Billings. Lincoln, Nebraska.

IV. Der Stabcher rothlauf und die Schweineseuche von Prof. E. Hess in Bern Thiermedizinische Vortrage, Heft 1–6, Band I. (Bacillary Erysipelas and Hog-plague). Halle, 1888.

V. Report of U. S. Board of Inquiry concerning Epizootic Diseases among Swine. Bureau of Animal Industry, 1889.

Seldom has the journalist so difficult a task placed before him as the review of the above-mentioned monographs and reports. A regrettable element of personality tinctures and impairs the value of at least one of these productions ; official incompetency and insincerity on the other side place its advocates in a much more unenviable light ; and worse than all, the umpires, if we may so designate them, and the high character and scientific standing of at least one of whom caused us to suspend our own judgment and await their decision, have failed to give anything like a clear and unmistakable opinion on the merits of the case. We are therefore compelled to briefly review the history of the dispute concerning hog cholera in this country before proceeding to a detailed review of this very much confused question. One of our most important governmental departments—that of Agriculture—has not at all times in its history enjoyed public confidence. For at least one Presidential term it was under the management of a commissioner, whose honesty we were inclined to question, until it occurred to us that his unaccountable acts and wild projects were the outgrowth of a peculiar mental organization which should have rendered him an object of sympathy rather than of criminal prosecution ; but, and this is the important point to the public, which should also have prevented his appointment to a responsible position, where his capacity for doing harm was so great; is it to be wondered at, that under such mismanagement our Governmental Reports on cattle plagues should become the laughing stock of experts? Have we not reason to congratulate ourselves on the fact that these reports are seldom read? Look at this passage from the Report for 1880, anent the so-called "Swine-Plague" in the South-West :—*

* The italics are our own.

"With from one to 300 diameters I find a dark and nucleated cell or cells with dark outline and dark centre. The cell appears circular at first, but in its different stages of *aggregation or fermentation*, it assumes an egg-shape from the piling of one upon another; or a better comparison would be that of a cauliflower or head of curled lettuce. The tissues of these cells appear ecchymosed. A red globule *seldom appears* after death.* but yellow serum and dark grumous blood are generally found. The blood before death is pale and watery. A drop shows one-half yellow water or serum, and the other half a disorganized clot of a purplish cast. This was particularly noticeable in a cow that died *of charbon and hog cholera combined*. I suggest the name of putrid measles for this disease, as I think it describes better the character of the malady, and will be more satisfactory to the profession. In some districts it should be designated malignant or black measles, which is *not* considered so fatal."

Can one remain serious on reading such a delirious jumble? Is the reviewer, who in noticing the above at the time of its appearance, unjust,—howbeit his mode of expression was undignified—when commiserating the author about the loss of three mules by a disease which is alternately called measles, swine-plague, amalgamated hog cholera and charbon, and whose malignant form is not as fatal as the benign form, he expresses a hope that his wife's mule** may survive, but on condition that he write no more?

It is simple justice to say that even at this inauspicious period in the history of Agricultural Department pathology valuable reports by Law, Detmers, and others were published. But in the main, the volumes of 1880–1883 consist of scattering records from here and there, of abstracts of papers published in foreign journals, and singularly enough of "auto-reviews," if we can coin a word to define a review, in its own "Special Report, No. 12," which this Department of Agriculture indulged in. Regarding this review, an indignant and amused colleague called our attention to the fact that it was chiefly directed against Dr. Detmers, who had the good or bad fortune not to be a government pensioner at the time. That veterinarian had published certain good results obtained in hog cholera through the rational and systematic use of carbolic acid. The department pensioner and expert, however, scouts this, and suggests that swill feeding, because (if associated with corrosive sublimate) it sweeps the *bacillus suis* out of the bowels, will

* Probably because it is discouraged under the circumstances, or shrinks from such immortalization in the Agricultural Reports.

** Here is another extract: "Two of my hogs were lousy, and in a bad state generally, bedded with the boar and took the disease. With the constant use of chloride of ammonia I kept one alive for two months, and I feel that this had a beneficial effect on the lung and throat trouble that generally accompanies this disease. Some had sore and swoolen noses, and four sucking pigs had an eruption resembling chicken-pox. If this eruption could be kept out I believe it would prove beneficial to the animal. I have used coal oil and turpentine with marked effect, a teaspoonful of turpentine, or a tablespoonful of coal oil in a little gruel. I have also used caustic, potash and concentrated lye." Further on the gifted author refers the contagious element to horse-flies, and evolves the following glorious specimen of entomology: "In the mornings and evenings we have swarms of a small yellow fly, which are very troublesome. Whether these are young horse-flies or not I am unable to say. Then we have the large horse-flies with green or striped head, and a smaller one that stings badly. There is still another which we call a cattle-fly—an extra large black fellow that looks much like a hearse or death carrier."

Under ordinary circumstances a man who sees a cell fermenting into an egg-shape, to assume a cauliflower and lettuce shape with his microscope, and flies of progressive malignancy in shape, size and color, flitting about, the bearers of doom, with his naked eye, would not present a very difficult case for diagnosis and treatment at any fairly well managed hospital.

prove more effectual. He then proposes to give all hogs a bath of a weak solution of bichloride of mercury, to pour a similar solution down their throats, and to feed them on eggs. Probably the "common people" who require "only the leading facts," as this report correctly says, can surmise how many dead hogs would receive a bath, when a herd of three or four hundred are effected, and how many eggs would be eaten. A little later the Department of Agriculture lent itself to a very questionable aim, in furthering, or at least tacitly endorsing, a crusade against Dr. Detmers, when he was accused of having falsely started a pleuro-pneumonia scare, and it was demonstrated beyond peradventure by the British Vice-Consul, Mr. Crump, that it saw fit to conceal the existence of hog cholera in the interest of pork exporters, thus either contradicting its own earlier and later reports, or tacitly admitting that it had allowed its pages to be crammed with manufactured statistics and bogus investigations.

The present controversy as to hog cholera is largely the outgrowth of researches conducted by Frank S. Billings, who, provided with a laboratory by the Nebraska State authorities, devoted himself to studying the subject with equal ability and enthusiasm. We would rather not be compelled to say that while the former quality enabled him to discover the truth and to correct errors, the latter has taken the shape of vehemence in personal accusation. It must be admitted that Dr. Billings, if he has learned the lesson, that particularly with the American medical reading public, it is not always wise to call a spade a spade, certainly does not practice it in the third pamphlet enumerated above. The editors of the medical press (some of them, we believe he intended to say) are described as "erudite old fogies and monuments of conceited respectability." Many of us may have had these very words on the tip of the tongue, but we believe few would venture to print such thoughts. It may be "cowardly" as Dr. Billings says, to admit this, but the position we hold relieves us of the necessity of making such martial and high sounding declarations as this; "Not being a coward, and fearing no power this side the grave, or beyond it, I propose to enter at once on this disagreeable task," or page 6, "Let him who dares say it is a 'personal fight.' I deny it! It is simply the fulfillment of my obligations to my profession and the live stock interests of my country, and my race in all countries, whose true and devoted servant I am endeavoring to be." Such pronunciamentos are not regarded favorably to-day, although we believe the moral sense of the community is not lower than it ever was. The orators of the French Revolution used such language, and were in the habit of figuratively throwing themselves on their country, and allowing themselves to be immolated on the Altar of Liberty, just so Dr. Billings heroically defies the Department of Agriculture, wrapped in the mantle of his innocence, scientific pride, and zealous honesty, and is prepared to fall back on the altar of the cattle interests of his country, a martyr to bacteriological investigation. We have a right to be indignant with Dr. Billings for mutilating his useful work by such a farrago, as we shall proceed to show him later on.

We have compared the extracts cited by Dr. Billings from the reports of the Department of Agriculture, line by line and word for word; we have carefully examined the plates accompanying these reports to which he refers, and we have also read the entire context from which he quotes, in order to avoid any possible bias, which may follow from reading disconnected sentences. As a result we are enabled to say that all of Dr. Billing's charges, sweeping and severe as they are, are true and just, and we can not understand how the committee appointed to investigate the special question involved, could fail to make the same examination, to arrive at the same result, and to publish that result in calm judicious language, not as a matter of justice to Dr. Billings, or of criticism of Dr. Salmon, but as a positive duty to science. Regarded from this point of view, the report of that committee is the most disappointing document of the kind we have ever seen. This we may say without intimating, as Dr. Billings does, that its authors were under the control of the "Bureaucratic Whip."

The charges made by Dr. Billings, expressed in more conventional language than he employs himself, are that Dr. Salmon's bacteriological work is not expert, that he described a germ of swine plague when he had no such germ in his possession, that he has printed assertions, without scientific evidence to sustain them. He even states directly that one micro-organism described by Dr. Salmon was evolved from the inner consciousness of that gentleman, and designates this procedure by a term to be found in the criminal code, and not usually employed in scientific discussion, however applicable it might be regarded in one sense to evidence, which Dr. Billings states to have been deliberately made up for the occasion.

Regarding the bacteriological questions involved, we would be inclined to suspend judgment, were it not for two sets of facts. First, the evasions of the "Board of Inquiry;" second, the gross logical errors and inherent contradictions of Salmon's writings; the latter are glaring, it would not have hurt Dr. Salmon's reputation, had he on being convinced of errors in his methods or conclusions, candidly acknowledged them, and accepted the corrections furnished by others and verified by himself. Men of a real rank, in the scientific hierarchy like Virchow, Klein, Koch and others,—and Dr. Billings here sets his opponent an example worth imitating—( No. III, pp. 50–53)—have not hesitated to acknowledge mistakes, such as the most honest and able investigators are liable to in experimental science, as well as in interpreting or quoting the results of others. But Dr. Salmon has preferred to gloss over the matter, and in order to sustain a claim to priority at the expense of another, and to conceal the fact that he adopted the corrections of others, has involved himself in a labyrinth of contradictions; the inevitable consequence of misrepresentations, be they intentional or unintentional. Which of the two classes Dr. Salmon's belong to, no one who reads Dr. Billing's pamphlet and verifies its citations can for a moment doubt.

Briefly, the history of hog cholera in the United States, is this: In 1878, the swine breeders became alarmed at the great losses sustained by an epizootic disease, and the Government appointed two veterinarians, Detmers and Salmon to investigate the subject. Detmers discovered a germ the *bacillus suis* which is now generally recognized, and first announced the theory that the epizootic in question is a germ disease. In the report of 1880-81, Dr. Salmon announces a micrococcus as the cause of the swine-plague, and repeated references to the same are to be found in various reports down to 1885. In the report above mentioned, dated 1889, the germ is spoken of as the hog cholera bacillus. In connection with these facts, the following admission made by Salmon has a very strange appearance. In speaking of the association of Dr. Smith in his work, he says that he made some slight changes in the latter's report: a phrase reading "we no longer consider a micrococcus as the cause of swine-plague," to "we no longer consider a micrococcus as the cause of all outbreaks of the disease known as swine-plague." Now it is only too evident that by a sinister coincidence, while Dr. Salmon was preparing to abandon the micrococcus and return to the bacillus (without any disfiguring reference to the real discoverer) he claimed the existence of two diseases among swine, calling one "hog cholera," and the other "swine-plague." Through all these gyrations and évasions Dr. Billings hunted Salmon with the keenness of a medical Nimrod, and we think proves that if Salmon's procedure was not, as he intimates evasive shuffling, that he has manifested a most remarkable kind of pathological knowledge, one altogether unprecedented in our reading. Eventually, Dr. Billings induced Congress to appropriate $30,000 to defray the expenses of an investigation into the disputed points. Dr. E. O. Shakespeare, Prof. J. V. Burrill and Prof. B. M. Bolton were appointed a committee of investigation. Now, although there was a great difference of opinion about vital points between Dr. Salmon and Dr. Billings, to whose exertions the appointment of the committee was due, what will the reader say on finding that the report passed through the hands of the former, one of the parties to the controversy. It does not look like fair dealing.

The report unfortunately, and reluctantly we say it, in view of the very high esteem in which we in common with others, hold the eminent pathologist of the committee which made it, betrays its nature in almost every line as a whitewashing report. It does not venture to touch on most of the disputed points. The one vital issue it discusses, it discusses as Salmon's mouth-piece, pure and simple. That in adopting this position, the committee should experience its natural results, is simply retributive justice. They say, "these two epidemic diseases* have been fairly well described in the recent annual reports of the Board of Animal Industry, except, it does not appear that the hog cholera of these reports can be said to have its special or exclusive seat in the digestive tract of the animal as distinct from the lungs." What to say of this stupendous insult to the average pathological understanding, we do not know. We submit that we are unable to characterize it. Suppose that a commission were to report on the claim of a pathologist that typhoid and intermittent were two distinct diseases,** and

*Meaning the two forms which Salmon claims and Billings repudiates.

**It is not necessary to point out how much worse the committee's quandary becomes if it turns out—as seems probable—that Billings, already endorsed by distinguished foreign authority, is right in considering but one disease.

endorsing the distinctness, say "except that the intermittent fever of these reports can not be said to have its special or exclusive seat in the heat center as distinct from the spleen and blood."

Regarding the bacteriological side of the question, the commission is equally unfortunate, and although the majority report claims that on the substantial point a dissenting minority report by Professor Bolton is in accord with their own, we do not think our readers will agree with them. Prof. Bolton says:

"During my work as commissioner I have failed to meet with an epizootic which I am satisfied was what is termed "swine-plague" in the Bureau reports, though previous to my appointment on the Board I studied one such outbreak. In this case, however, I directed my attention to the bacteriological questions exclusively, and I am therefore unable to pronounce on the difference in the pathological lesions in the two diseases. But I am not inclined to attach any great importance to these differences as set forth in the reports. The description otherwise I find correct and well stated. In my investigations as commissioner I have been able to find but one organism which in my opinion caused the outbreaks under examination, and that I regard as identical with the hog cholera germ described in the reports of the Bureau, and I find the description therein given correct. As will be inferred from what has gone before, I feel sure that another organism, correctly described in the reports as the "swine-plague germ" is found under circumstances which render it highly probable, if not certain, that it also causes disease. As to whether these two organisms are always present and operate together to cause disease, or whether the two are merely varieties of the same germ, must be decided by future investigation."

The main report insists that there are at least two wide-spread distinct diseases which are caused by distinct micro-organisms. If this is what the committee calls agreeing, we would like to know what disagreement is. Who will be in doubt for a moment as to who, or what induced Prof. Bolton to write the last few lines of his report. They are merely loop-holes for the escape of the man, through whose hands the report went. Whether pity or courtesy were Professor Bolton's motives, he owed a higher duty to the country, than to Dr. Salmon; we regret that he was not less ambiguous in expressing his convictions, although as contrasted with the majority report, we have occasion to be grateful for even this much.

One of the latest publications on that subject, the monograph of Hess, (iv) written without any bias, and evidently without any knowledge of the bitter personal nature of the controversy, has the following: According to the papers of Klein it seems natural to adopt the view, which, bye the bye, has found acceptance in the most recent text books, that Klein's infectious pneumo-enteritis is to be classified either as bacillary erysipelas or swine-plague; chiefly under the latter head, because Klein lays so much stress on the pneumonic symptoms. Baumgarten also inclines to the view that what Klein identified as erysipelas, is not bacillary erysipelas, but rather swine plague. The disease observed by Detmers in Chicago, 1877–78, and described as swine-plague, hog fever or hog cholera, which appeared in a very virulent form at the above date, and caused a mortality of 75 per cent., is according to the most recent researches of Salmon not identical with bacillary erysipelas but with swine-plague. *It is very probable that the* disease described by

Detmers is the same as the one described also by Klein. The North American hogs also showed symptoms of pneumonia, pleurisy, and pericarditis, as well as of peritonitis and enteritis prominently developed. The disease germ described by Detmers as *bacillus suis* does not resemble *the erysipelas bacillus*, but has more likeness to the *bacterium* of Koch, Gaffky's rabbit septicæmia.

After discussing the contradicting and uncertain results of Pasteur, Hess summarizes the conclusions of the able researches conducted by Löffler, Schütz, Lydtin, Shottelnis and Cornevin, by stating that they show the existence of two distinct diseases among swine, hitherto confounded, of which one is the true swine erysipelas (bacillary erysipelas), and the other is the swine-plague. The bacilli of the former are rod-shaped 0.6—1.8 micro-millimeters in length, and found singly or associated among (between) the red blood globules, or enclosed within the white ones. Probably their latter location would be more frequently patent to the observer, if they did not cause a disintegration of the matrix leucoyte. Protective inoculation has been proposed and carried out by Pasteur. Several (Lydtin) have confirmed his favorable results, while Hess thinks that the results are not yet convincing enough to justify this method's adoption.

The bacillus of swine-plague (really discovered but imperfectly described by Detmers * in 1879) was accurately studied by Löffler in 1882, and later by Schütz, and is ovoid 1.2 micro-millimeters long, and 0:4–0.5 broad. They only stain at the poles, the middle unstained part being the one in which fissure occurs in proliferation.

The "Œsterreichische *Monatschrift fur Theirheilkunde*," No. 4, 1889, contains excellent illustrations of the micro-organisms of both diseases, and the authors furnishing them sufficiently manifest their appreciation of Salmon's efforts to make different diseases of swine-plague and hog cholera, by the following designations of this figure V: Schweinepest (hog cholera). It is evident that considerable dust has been thrown in the eyes of European writers by the report of the Department of Animal Industry, and like the authors just cited they seem to have been as hopelessly bewildered as we have been in attempting to reconcile their conflicting descriptions, conclusions and nomenclature.

On carefully reviewing the present state of our knowledge on the subject of American swine-plague, it appears evident to us, 1st, that Salmon's micrococcus is not and never was a specific disease-germ of anything. Whether created in the manner suggested by Billings, or due to the adoption of erroneous methods, it will disappear, if it has not already disappeared from the columns of trustworthy bacteriological literature. 2d. That while Salmon in 1886 claimed swine-plague to be a distinct specific disease, characterized by pneumonia, his hog cholera was described as an equally specific disease characterized by an equally characteristic enteritis, he totally abandoned this position in 1887.

Now it seems to us to have been the patent duty of the commission

* And badly photographed by him, see Agricultural Report for 1880–81.

appointed, to determine these very questions, to discover what proof Salmon offered in favor of his first position, and what proof induced him to abandon it. This they have not done. They have given in return for a generous appropriation not a single position or useful decision, and no valuable result. The only practical issue considered by them is that of preventive inoculation, as suggested and carried out by Billings in Nebraska. This procedure is "damned with faint praise" or worse; for the commission goes out of its way in sounding an alarm against the whole principle of protective inoculation, in which it may or may not be right, but unfortunately has omitted to accept Dr. Billing's offer to demonstrate his claims, and passed upon them in an indirect, evasive way without any fair examination.* We regret to add that in special reference to Dr. Billings, the committee manifested a quibbling spirit, which is in singular contrast with the liberal construction made of the gross ommissions and inaccuracies of Salmon. They criticise Billings for not having obtained his cultures from the lungs, intimating that Salmon's second germ would have been found in that notorious sewer trap. That Billings obtained no cultures from the lungs, is nowhere apparent, but we thoroughly endorse him in his very proper claim, that the engorged capillaries would be certain to furnish pure cultures. In their anxiety to discredit Billings, the commission overreached themselves. One objection they make to the protective inoculations practised by him, is that the inoculated swine may re-infect the earth from their fæces. Admitting this to be true, it is a vital point against Salmon, who claimed the disease to be contagious. They thus in attempting to invalidate Billing's results in one direction, unconsciously sustain him in another.

Otherwise the committee report is lamentably defective in everything that could entitle it to be regarded as a scientific production. The assertions—for they are not results—we have reviewed above. Aside from these, there is not a single autopsy reported, nor the details of a single experiment. In speaking of "a wide-spread epidemic" (they probably mean "epizootic") diseases of hogs, they do not tell us in what and in how many states they exist, where the limits are, whether or not and where they run into each other, in short they give not a single one of numerous important facts that could have been learned, by an earnest investigation on the spot—for which the committee was generously salaried—or by an intelligent collating of reliable evidence by others. In the face of all this their admission that the Salmon swine-plague is not quite as prevalent as the regular thing, is self-convicting. If this commission be continued, we have no doubt the Salmon swine-plague will grow beautifully less in a year or so, gradually dying out, thus giving the head of the Bureau a chance to "let himself down easy." Nay, there is not the slightest reason in the world, why Salmon might not turn Billing's artillery against the latter, and prove by such a commission that he had stamped out one of the plagues through his able management of the Bureau. The bacteriological historian would then have reasons to

*If the evidence of stock breeders be worth anything, Dr. Billing's claims are entitled to renewed and earnest examination; for they seem to be strongly in his favor.

rejoice that Salmon's micrococcus, however non-existent, at a later day, was embalmed in the very nick of time in the annual reports aforesaid.

Some inoculated hogs were placed at the disposal of the committee by Dr. Billings. The former report that these hogs withstood every test they were put to, but they do not say what "tests." Now this is precisely what the stock-breeder ought to know. Here is a governmental investigation! Samples of what is claimed to be well-protected hogs are plac d at their disposal; it might have been expected at the very least that detailed and exclusive tests should have been made to determine so vitally important a question.

The work done by Billings at Lincoln, is of the highest character, and his record as an original discoverer of *real* (not fictitious) disease germs is so much above that of any blind follower of other investigators, that his claims were and are entitled to more than a perfunctory analysis. He has not been the only critic of Salmon's imperfect methods; Seiler, of Philadelphia, has made similar criticisms. The discoverer of the Texas fever germs, and the germ of "corn-disease," did a good service when he exposed the bad work of the Bureau of Animal Industry. And feeling that the exposure was necessary, and might have p oven effective in securing a competent head for that bureau had Dr. Billings contented himself with calm matter of fact criticism, and manifested a little patience, we have some grounds to censure him for spoiling the good work by invective and feverish anxiety. His opponent has now, through Dr. Billing's injudiciousness, an opportunity to evade the discussion, on the ground of the character Dr. Billings gave it. To the charge that he discovered a micrococcus, where there was none, Dr. Salmon may answer that the one making the charge called him names. To the charge that Dr. Salmon raised an unfounded scare about the hog plagues when there was but one—and that one bad enough—he may answer that Dr. Billings used extravagant and ill-tempered language in opening that question. It is true that no good man, and no reflecting man would regard such a procedure as refutation. But the people who have the power of making government appointments are not all good men, and the public which is responsible for their election, is not always reflective. The former are willingly, the latter unconsciously duped by common-place cant and *bogus* authority. The labor of the reformer against these elements, has always been Sisyphus like, and if Dr. Billings should have failed in his efforts—which in the interests of the cattle industries of this land we sincerely wish well to—whom has he to blame for handicapping himself? ***

---

CONTRIBUTIONS TO THE NATURAL HISTORY OF THE CETACEANS. A REVIEW OF THE FAMILY DELPHINIDÆ. By FREDERICK W. TRUE. Bulletin U. S. Nat. Museum, No. 36, Washington, 1889.

This admirable monograph devoted to the natural history of the dolphins has just appeared as No. 36, of the series of "Bulletins" of the U. S. National Museum, and will undoubtedly at once fill a place that has long awaited just such a contribution to our working library in the fields of cetology. It constitutes a volume of some 200 pp., and is handsomely illustrated by nearly fifty highly useful plates. These latter are devoted to presenting from one to five or six figures of the species representing some nineteen or twenty of the genera of the *Delphinidæ*. In general each plate offers us a good figure of the lateral view of one of the group under consideration, with below it, an additional drawing of the base of the skull of the same species, a very excellent combination.

Other desirable features of this memoir are seen in its brief but eminently scientific "Introduction;" in its two "Keys" to the Genera, one being based upon external characters, and one upon cranial characters; and in its good systematic and general indices. Primarily, the book is divided in two parts, the first of which has to do with a "review of the species," and the second with a "synopsis of the species," and under this arrangement, Mr. True deals with sixty-two species of these dolphins, which are arrayed here under two subfamilies, and are created to contain seventeen and two genera, respectively. The two subfamilies referred to, are the *Delphininæ* and the *Delphinapterinæ*.

Throughout his labors, as our author candidly confesses, his work "has been directed not at all towards the distinction of genera, but rather toward the determination of species" (p. 6). And as heretofore, the species of this group have, for the most part, been very unsatisfactorily defined, a more opportune task could hardly have been accomplished; and science is fortunate in the fact that Professor Flower of the British Museum has under his hand a nearly completed work, which will more directly deal with the generic characters of these cetaceans.

A number of years ago, Mr. True became dissatisfied with the insufficient descriptions which described our North American species of dolphins in literature, and prompted by this he undertook to re-examine all the type material in the premises, and even visited Europe and its museums to the same end, the volume here reviewed being the outcome of these important researches. In order to appreciate just how meagre the literature is in some cases, I quote our author from his preface, and he says "The genera *Orca* and *Orcella* are not touched upon in this paper. The species of the latter genus need no elucidation. In the case of *Orca*, the material which I gathered is scanty, and I abstain from discussing it for fear of adding to, rather than lessening, the confusion in which the genus is involved. Many additional facts must be obtained before even a tolerably satisfactory account of the killers can be written." In the case of the species described, however, our authority has in no instance, apparently, neglected a single factor that is demanded by science from the naturalist in so far as each specific description is concerned, and the scope of the work calls for. They are clear, brief, and to the point, being accompanied by a short bibliographical notice in each case, the whole being brought up abreast with our present knowledge of these matters. Mr. True is to be congratulated upon the appearance of his classical production; science is to be congratulated upon this valuable addition to her literature, and working naturalists for so useful a volume; while finally, the National Museum is to be congratulated upon being enabled to thus add another distinguished monograph to her already fine series of "Bulletins," and that, too, a work which although it deals strictly with the *species* of a certain group of mammals, still shows in its pages an appreciation of the value of a knowledge of the *structure* of the same, a fact indeed most praiseworthy, as in most quarters, even in this country, it is being conceded that the day is far advanced when we should pass from the mere "descriptions of species" to the higher work,—a dealing with their morphology. R. W. S.

INEBRIETY, ITS ETIOLOGY. PATHOLOGY, TREATMENT AND JURISPRUDENCE. By NORMAN KERR, M.D., F.L.S., Fellow of the Medical Society of London, etc., etc. Second edition. H. K. Lewis, 136 Gower Street, W. C., 1889.

This valuable treatise has already acquired a wide-spread reputation through its first edition and comes to us again handsomely recast, enlarged, and improved in the shape of a second edition.

Dr. Kerr struck the true key-note of drunkenness when he characterized it as constituting a special morbid condition depending on an abnormal diathesis. This accounts for the fact that so many can use liquor in moderation (whether beneficially or not is not here the question) without developing that insatiable craving for it to be witnessed in those who possess the true narcomania. Temperance agitators have failed, as a rule, to note this distinction, and the following consequences have resulted from their oversight. In the first place since all are usually warned against the bad effects of alcohol, and an equal liability to its horrors are predicated of all, those who are conscious of their complete immunity from its dangers, are disposed to treat the appeals of temperance advocates as the expression of an unreasoning fanaticism. So far, therefore, this oversight has operated harshly against the well-meant efforts of our lay temperance advocates. But it is especially in regard to those who are the victims of this unconquerable appetite, that the disregard of its pathological character has been fraught with mischief. Drunkenness has been depicted as an awful crime and its victims pointed out as the arch enemies of society. Their frequent lapses have been treated as so many voluntary returns to sin, and while the poor wretches themselves shuddered at their misery and deplored the tyranny that enslaved them, the "unco gude" reformers hold them up as awful examples of human depravity and incorrigible viciousness. Thus the excellent intentions of the prohibitionists have often been brought to nought by their unwise and indiscriminate denunciations of all who either traffic in or employ alcoholic beverages. Dr. Kerr has shown himself both wiser and more human than the rank and file of his followers in dealing with drunkenness as a disease to which persons possessing certain constitutional tendencies and weaknesses are especially prone. Thus the question of legislation so far as it affects the manufacture of intoxicants and the status of alcoholists, becomes narrowed to its proper dimensions, and so becomes greatly simplified. Of course, this pathological view of alcoholism does not by any means totally eliminate the element of volition from drunkenness, but rather accentuates the danger to which those are exposed who possess the narcotic heredity, and augments the moral necessity under which they are placed of absolute and unqualified abstinence.

In the chapter of the "etiology of inebriety," Dr. Kerr deals ably and exhaustively with this burning question, and leaves the impression on the mind of his readers that even where no danger exists for the many, they still are infinititely better off without alcohol than even when are employing it in physiological moderation.

On the whole Dr. Kerr's book must be regarded as the most valuable contribution to the literature of temperance that has ever emanated from the English press.

